# Comparison of early F-18 Florbetaben PET/CT to Tc-99m ECD SPECT using voxel, regional, and network analysis

**DOI:** 10.1038/s41598-021-95808-8

**Published:** 2021-08-18

**Authors:** Soo Jin Kwon, Seunggyun Ha, Sang-Won Yoo, Na-Young Shin, Joo Hyun O, Ie Ryung Yoo, Joong-Seok Kim

**Affiliations:** 1 Division of Nuclear Medicine, Department of Radiology, Seoul St. Mary′s Hospital, College of Medicine, The Catholic University of Korea, 222, Banpo-daero, Seocho-gu, Seoul, 06591 Republic of Korea; 2 Department of Neurology, College of Medicine, The Catholic University of Korea, Seoul, Republic of Korea; 3 Department of Radiology, College of Medicine, The Catholic University of Korea, Seoul, Republic of Korea

**Keywords:** Molecular medicine, Neurology, Computational neuroscience, Image processing

## Abstract

This study aimed to validate early-phase F-18 Florbetaben positron emission tomography (eFBB PET) as a brain perfusion test and determine the optimal reference region. A total of 27 patients with early Parkinson’s disease with Tc-99m ethyl cysteinate dimer single photon emission tomography (ECD SPECT) and FBB PET were included. Six reference regions, including whole brain (GN), pons, central white matter (CWM), whole cerebellum (WC), WC with brain stem (WC + B), and cerebellar grey matter (CG), were applied to obtain SUVR using cortex volume-of-interest (VOI). Reference regions of WC (*r* 0.886), WC + B (*r* 0.897), and CG (*r* 0.904) had highest correlation values of cortex-VOI SUVR between both perfusion images (all *p* < 0.001). Early-phase FBB PET had a significant linear correlation of CG-normalized SUVR of the cortex, basal ganglia, thalamus, and midbrain with ECD SPECT in voxel-wise analysis (FDR adjusted-*p* < 0.05). Early-phase FBB PET extracts more ICNS than ECD SPECT, as 9 ICNS and 4 ICNs, respectively. Both eFBB PET and ECD SPECT well discriminated PD from DLB (Area-under-curve of receiver-operating-characteristics, 0.911 for eFBB PET, 0.922 for ECD SPECT). Our findings suggest that eFBB PET is a reliable perfusion test based on a high correlation with ECD SPECT using cerebellum-based normalization methods.

## Introduction

Most neurodegenerative diseases are characterized by progressive accumulation of misfolded proteins causing neuronal injuries and are now recognized as having spectrum disease entity of mixed-proteinopathies of amyloid-beta (Aβ), tau, or alpha-synuclein^1–4^. The overlapping of the clinical and pathologic features of neurodegenerative disease underlines the demand for a multimodal diagnostic imaging approach such as amyloid positron emission tomography (PET), fluorodeoxyglucose (FDG) PET, and magnetic resonance imaging (MRI). A combination of amyloid and FDG PET imaging can support a diagnosis of the neurodegenerative diseases, differentiating Alzheimer’s dementia (AD) and dementia of Lewy body, which are the first and second most common dementia. However, those approaches have limitations in routine clinical settings due to the cost and radiation hazards.

Considering the perfusion-metabolism coupling, perfusion imaging is a substitute for FDG PET imaging to evaluate neuronal injury. Amyloid PET tracers, including C-11-labeled Pittsburg compound B (PiB), had a high first extraction rate, which may provide brain perfusion information from early PET imaging^5^. Therefore, dual-phase or dynamic amyloid PET imaging can be used to obtain the information of brain perfusion and amyloid burden at once. Regional standardized uptake value ratio (SUVR) derived from early-phase amyloid PET or regional kinetic parameters showed a moderate to high correlation with FDG PET^6–9^. However, there are only a few studies directly comparing early-phase amyloid PET imaging and validated perfusion imaging to date^10^.

The aim of the study was to conduct a head-to-head comparison of cortical SUVR from early-phase F-18 Florbetaben PET (eFBB PET) to a validated brain perfusion imaging of Tc-99m labeled ethyl cysteinate dimer single photon emission computed tomography (ECD SPECT) and to find optimal reference regions for perfusion assessment of eFBB PET.

## Results

### Demographics

**Figure 1:**
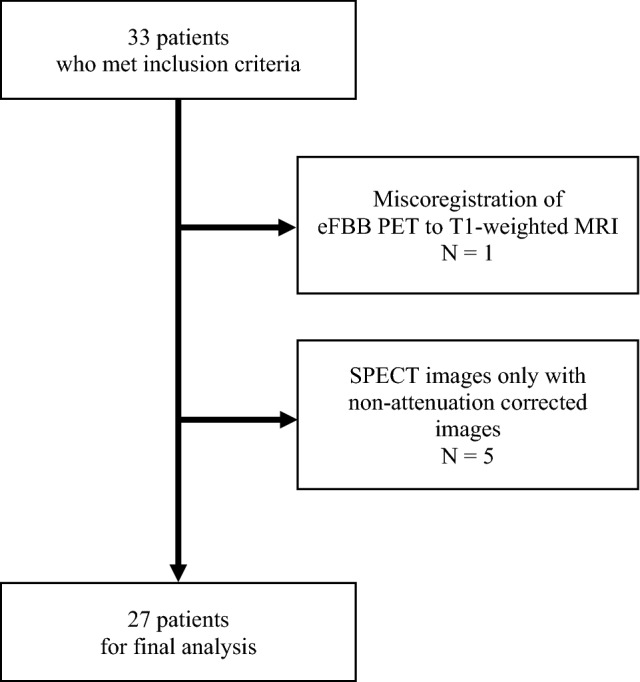
Flowchart for patient selection.

**Figure 2:**
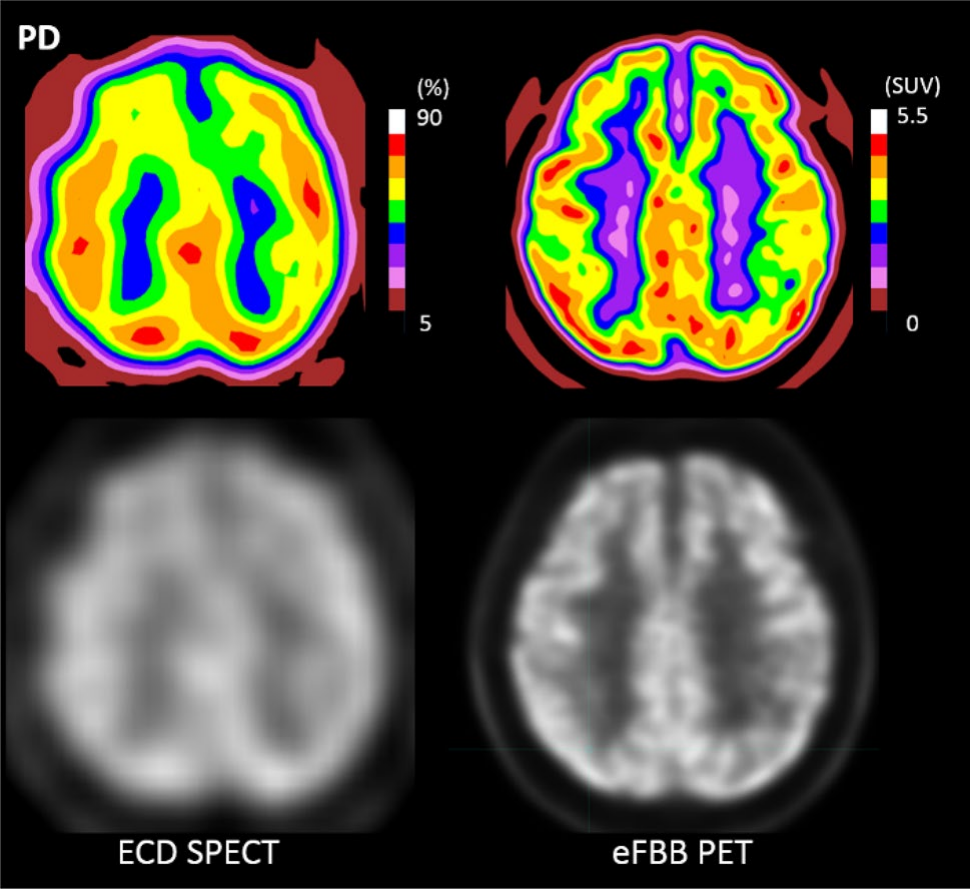
Sample images of ECD SPECT and eFBB PET scans of patients with PD. Sample images are visualized in 10-step colored images (upper row) and grey-scale images (lower row). Abbreviations ECD, Tc-99m ethyl cysteinate dimer; eFBB, early-phase F-18 Florbetaben; PD, Parkinson’s disease; PET, positron emission tomography; SPECT, single photon emission computed tomography; SUV, standardized uptake value.

A total of 33 patients met the inclusion criteria. One patient with misregistration of the eFBB PET image to the MRI image was excluded from the analysis. Five patients only with non-attenuation corrected SPECT images available in the database were also excluded. A total of 27 subjects (14 males, 52%) were included in the analysis (Fig. 1). The median age was 67 years (range 53–86 years). The median modified Hoehn–Yahr scale was 1.0 (range 1.0–2.5). Three late-phase FBB scans were visually interpreted as amyloid positive (Table 1). The median period between ECD SPECT and FBB PET was 2 days (range 1–26 days). A representative example of ECD SPECT and eFBB PET images from PD patients was presented in Fig. 2. Ten patients with DLB (5 males and 5 females) were additionally included to evaluate the diagnostic performance of two perfusion imaging modalities in comparison to PD patients. The median age of 10 patients with DLB was 79 years (range 71–88) (Supplementary Table 1).

**Table 1:** Characteristics of the study population.

	Values	Range
Age, years	67	53–76
**Gender**	Number (percentage)	
Male	14 (51.9%)	
Female	13 (47.1%)	
K-MMSE	28	23–30
UPDRS motor	16	5–42
UPDRS total	23	7–53
Modified Hoehn–Yahr	1	1–2.5

The values in the second column are median values if not specified.

*K-MMSE* Korean-Mini Mental Status Examination, *UPDRS* Unified Parkinson’s Disease Rating Scale.

### Correlation of cortical SUVR of early-phase FBB and ECD SPECT according to reference regions

**Figure 3:**
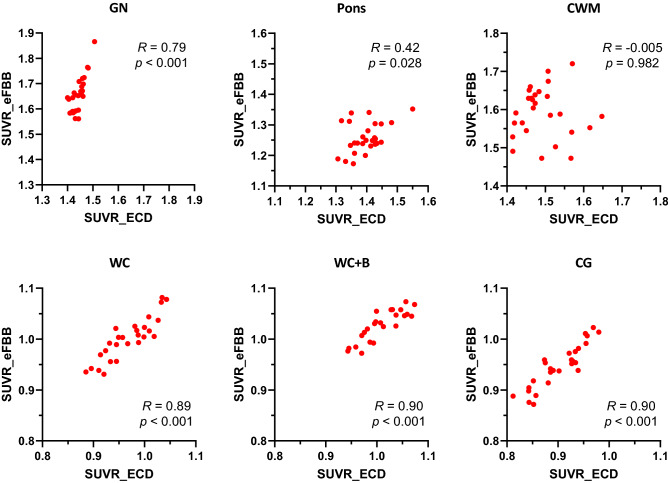
Correlation plots for the Centiloid cortex volume of interest-based SUVR of ECD SPECT versus eFBB PET based on the six reference regions. Pearson’s coefficient *r* and *p* values are shown on each plot. Abbreviations CG, cerebellar grey; ECD, Tc-99m ethyl cysteinate dimer single photon emission computed tomography; CWM, central white matter; eFBB, early-phase F-18 Florbetaben positron emission tomography; GN, Global normalization; SUVR standardized uptake value ratio; WC, whole cerebellum; WC + B, whole cerebellum and brain stem.

Correlation plots for the Centiloid cortex volume of interest (VOI)-based SUVR of ECD SPECT versus eFBB PET are depicted in Fig. 3. Most strong correlations were seen between two exams when normalized with cerebellum-based methods of whole cerebellum (WC), WC with brainstem (WC + B), and cerebellar grey matter (CG) as *r* of 0.886, 0.897, and 0.904, respectively (all *p* < 0.001). The global normalization method (GN) showed a high correlation coefficient but rather lower than the cerebellum-based normalizations (*r* = 0.791, *p* < 0.001). The pons-based normalization demonstrated moderate correlation (*r* = 0.42, *p* = 0.028). There was no correlation when normalized with central white matter (CWM) method (*r* =  − 0.005, *p* = 0.982). There was no significant difference in SUVR between the amyloid positive and negative groups.

### Voxel-wise comparison of early-phase FBB PET and ECD SPECT

**Figure 4:**
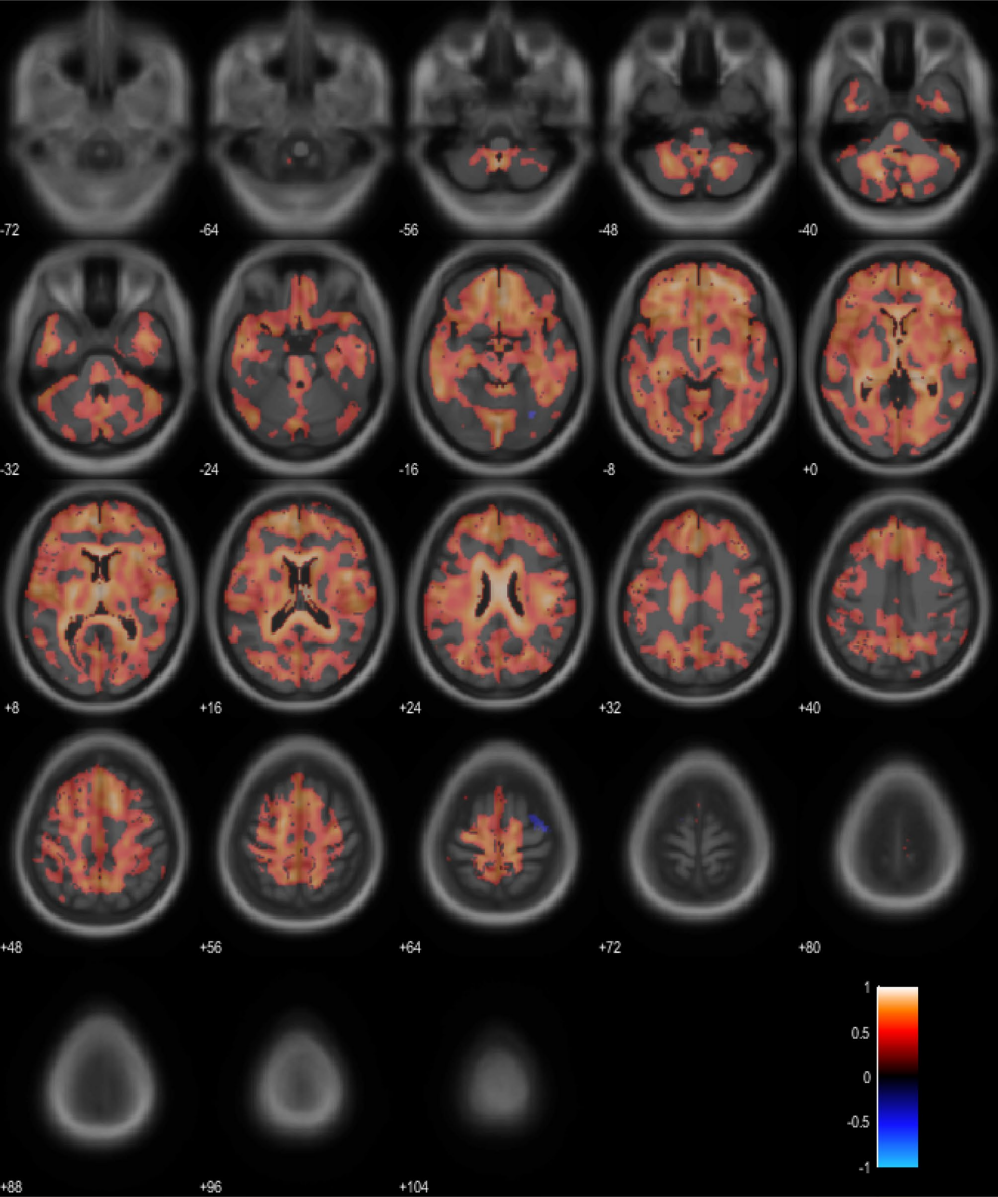
Voxel-wise correlation of eFBB PET and ECD SPECT with normalization by cerebellar grey (*p* < 0.05, FDR adjusted). The colored areas overlaid on the T1 weighted MRI template show voxels of significant linear correlation between SUVRs of eFBB PET and ECD SPECT (a). The color bar represents values of Pearson’s correlation coefficient. Abbreviations ECD SPECT, Tc-99m ethyl cysteinate dimer single photon emission computed tomography; eFBB PET, early-phase F-18 Florbetaben positron emission tomography; FDR, False discovery rate; MRI, magnetic resonance image; SUVR, standardized uptake value ratio.

Voxel-wise analysis was performed to compare the SUVR of eFBB PET and ECD SPECT for the CG normalization method. Most cerebral cortical regions, basal ganglia, thalamus, midbrain, and periventricular white matter show significant linear correlations between eFBB PET and ECD SPECT (False discovery rate (FDR)-adjusted *p* < 0.05) (Fig. 4). Early-phase FBB PET showed higher voxel-wise SUVR, mostly in cerebral cortical regions (frontal, parietal, temporal, and cingulate), striatum, thalamus, midbrain, and pons regions (FDR-adjusted *p* < 0.05). ECD SPECT showed higher voxel-wise SUVR in periventricular white matter region (FDR-adjusted *p* < 0.05) and cuneus region (Supplementary Fig. 1).

### Identification of the intrinsic connectivity network (ICN) in early-phase FBB PET and ECD SPECT

**Figure 5:**
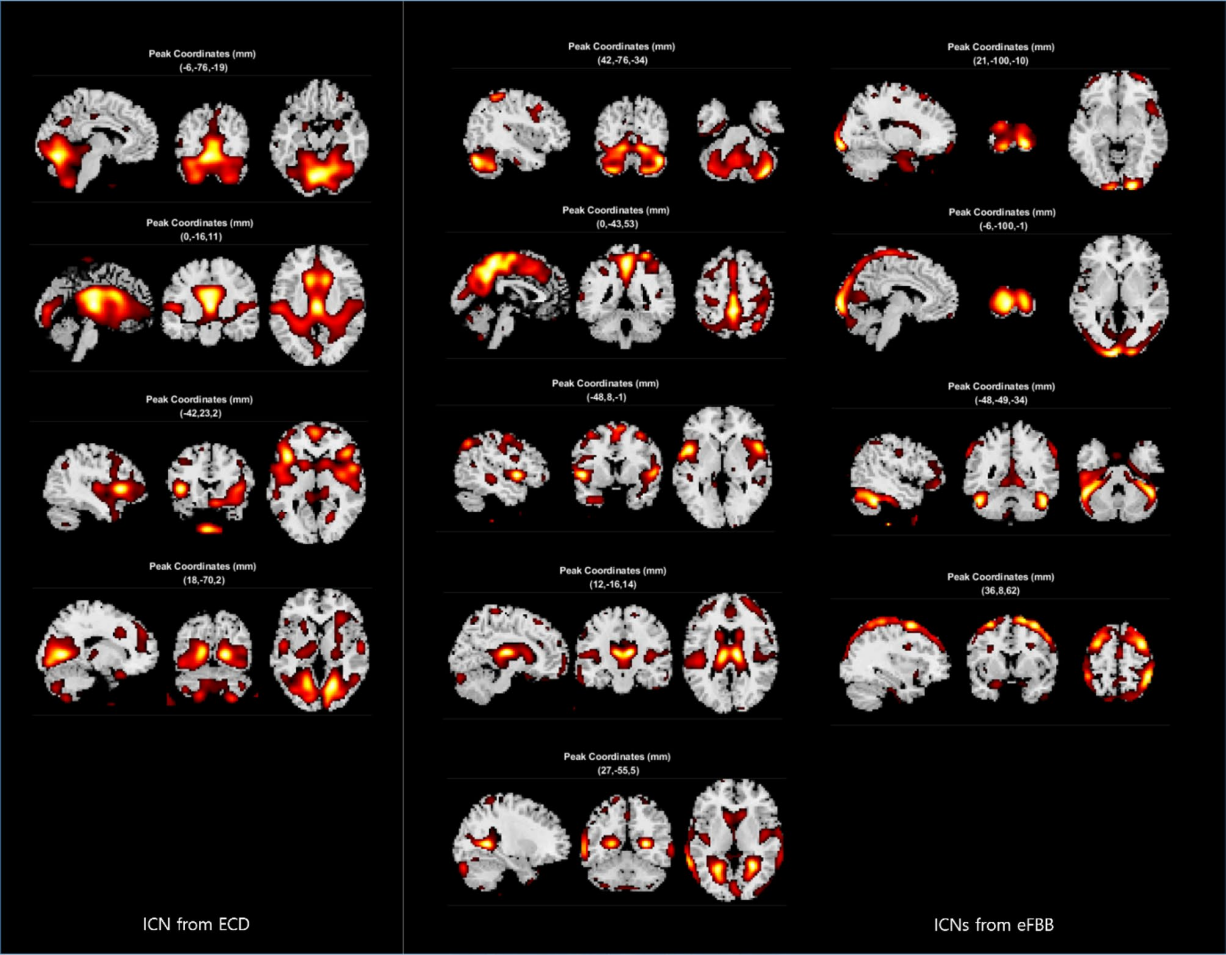
The intrinsic connectivity networks identified from ECD SPECT and eFBB PET. The images in the 1st column show the intrinsic connectivity networks derived from ECD SPECT, and the 2nd and 3rd columns from eFBB PET. Abbreviations ECD SPECT, Tc-99m ethyl cysteinate dimer single photon emission computed tomography; eFBB PET, early-phase F-18 Florbetaben positron emission tomography.

Total 4 and 7 intrinsic connectivity networks (ICNs) were extracted from CG-normalized ECD SPECT and eFBB PET, respectively (Fig. 5). In addition, from ECD SPECT and eFBB PET, 3 and 2 network patterns, considered as artifacts, were also extracted (Supplementary Fig. 2).

### Comparison of early-phase FBB PET and ECD SPECT in patients with DLB and PD

**Figure 6:**
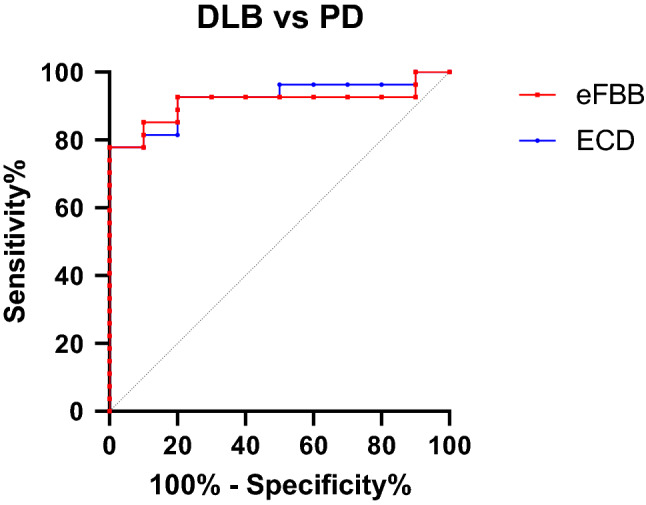
Discriminating power of perfusion imaging. The ROC curve for discriminating PD from DLB (AUC = 0.922 for ECD SPECT, 0.911 for eFBB PET). Abbreviations AUC, area under the curve; DLB, Dementia with Lewy body disease; ECD SPECT, Tc-99m ethyl cysteinate dimer single photon emission computed tomography; eFBB PET, early-phase F-18 Florbetaben positron emission tomography; PD, Parkinson’s disease; ROC, receiver operating characteristic.

Mann–Whitney U-test proved significantly lower perfusion of parietal, occipital, and insula cortices in CG-normalized ECD SPECT and parietal and insular cortices in CG-normalized eFBB PET (*p* < 0.05) (Supplementary Tables 2 and 3). We obtained the receiver operating characteristic (ROC) curves to evaluate the diagnostic performance of early-phase FBB PET and ECD SPECT for discriminating Dementia with Lewy body (DLB) from Parkinson’s disease (PD). The area under the curve (AUC) was 0.922 for ECD SPECT and 0.911 for eFBB PET (Fig. 6).

## Discussion

This study explored the optimal reference regions for comparison of cortical SUVRs of eFBB PET and ECD SPECT using a Centiloid-cortical-VOI. There was a strong cortical correlation between SUVRs of eFBB PET and ECD SPECT with cerebellum-based normalization methods. The GN method showed high but relatively lower than cerebellar-based normalized methods. There was a moderate correlation with the pons normalization method and no correlation with the CWM normalization method. Voxel-wise correlation test proved reliable correlation in the cortex, basal ganglia, thalamus, and midbrain between CG-normalized images of ECD SPECT and eFBB PET. More detailed ICNs were extracted from eFBB PET images than ECD SPECT images. Discrimination performance between DLB and PD was similar for CG-normalized images of ECD SPECT and eFBB PET. To the best of our knowledge, this was the first study to compare eFBB PET and ECD SPECT.

In our study, cerebellum-based intensity-normalization methods demonstrated stronger correlations compared to GN. Previous studies comparing kinetic parameters and SUVRs of early-phase amyloid and perfusion PET (H_2_O PET) showed a moderate to strong correlation with cerebellar normalization^10,11^. Meanwhile, a previous study comparing eFBB and FDG PET of patients with mild cognitive disorder and dementia showed a stronger correlation with GN methods, contrary to our results (*r* = 0.86 for GN and *r* = 0.76 for WC)^6^. The cerebellum is less susceptible to hypometabolism and deposition of misfolded proteins in neurodegenerative disease until late stages^12–14^. The cerebellar deposition of Aβ showed minimal impact on SUVR values of FBB PET even in the advanced stages of AD^15^. On the other hand, the whole brain as a reference region is vulnerable to a reduction of cerebral blood flow in neurodegenerative disease or normal aging. Global decrease of brain metabolism may also cause artifactual hyperperfusion in unaffected cerebral regions under the GN method^16^. We propose to use cerebellum-based references for these reasons.

For CWM reference, there was no regional correlation between eFBB PET and ECD SPECT. This finding is contrary to the previous study, which demonstrated a moderate to a strong correlation between SUVRs of early-phase florbetapir and H_2_O PET (range of *r*: from 0.70 to 0.86)^10^. The discrepancy between our results and the aforementioned results could be explained by the non-specific binding of amyloid tracer in subcortical white matter^17^. Our voxel-wise analysis also showed different distribution between two tracers throughout the white matter. The SUVR values of ECD SPECT were higher than eFBB PET in periventricular white matter. On the contrary, SUVRs of eFBB was higher than ECD in some part of centrum semiovale. The CWM is also susceptible to age-related ischemic changes. These indicate that CWM may not be the optimal reference for perfusion imaging of eFBB PET and ECD SPECT. Our study is limited by the difference in spatial resolution of the two studies and the absence of partial volume correction of SPECT images. Further studies are needed to validate white matter as an optimal reference.

The voxel-wise comparison demonstrated better grey-white matters contrast in eFBB PET than ECD SPECT. The SUVRs of cerebral cortices, basal ganglia, and brain stem were higher in eFBB PET, while the SUVRs of periventricular white matter were higher in ECD SPECT. The SUVRs in the outer edge of brain cortices seemed to be higher in ECD SPECT. This phenomenon might arise from the lower spatial resolution of the SPECT showing more blurred images compared to PET. Previous studies have reported that perfusion SPECT has the lower diagnostic performance to discriminate neurodegenerative diseases compared to FDG PET, which might be related to poor resolution and grey-to-white contrast^18,19^. The high grey-to-white contrast and high resolution of eFBB PET may be helpful to clinicians in the visual assessment of perfusion images to discriminate neurodegenerative diseases.

The ICA analysis revealed more ICNs from eFBB PET compared to ECD SPECT in the same cohort. The eFBB PET may also be more appropriate for functional network analysis. The difference in the number of ICN extractions is thought to be mainly due to the difference in resolution of the two images and the difference in grey-to-white matter contrast. However, further studies comparing eFBB PET to well-known metabolic imaging (e.g. FDG PET) are needed for validation.

We compared the eFBB PET and ECD SPECT of patients with PD to patients with DLB to explore the diagnostic value of eFBB PET. Our study demonstrated the comparable discriminative power of eFBB PET to ECD SPECT for DLB versus PD in the ROC analyses. The right insula was selected as the candidate feature based on the logistic regression with the forward propagation for both imaging modalities. Although not involved in the logistic model, SUVRs of the left insula were also significantly lower in DLB than PD. These findings are consistent with the previous study, which showed the lower cerebral blood flow measured from the Tc-99m labeled hexamethylpropyleneamineoxime (HMPAO) SPECT of bilateral temporo-insular regions in DLB compared to the PD group^20^. Several studies suggested that the hypometabolism in FDG PET or atrophy of the insula in MRI serves as the critical feature(s) in DLB, including prodromal stages^21,22^. Low perfusion to parietal lobes in DLB noted on both ECD SPECT and eFBB PET images were also concordant to previous results suggesting typical patterns of the cingulate island, which preserved cingulate metabolism with decreased parietal metabolism (Supplementary Tables 2 and 3)^23^. Further studies with a larger population of both PD and DLB are needed to confirm the diagnostic value of eFBB PET in clinical practice.

There are several limitations to this study. This was a retrospective study that included a small number of patients. The population included only early PD patients with mostly negative amyloid deposits. In this study, not only healthy controls but also the patient groups (AD or MCI) where FBB PET is the most used were omitted. However, there have been arising interests in the impact of proteinopathies on the cognitive disorder in PD patients^24,25^. Furthermore, the PD cognition-related pattern (PDCP), as well as PD motor-related pattern (PDRP), was elucidated based on FDG PET and perfusion studies^26^. These findings emphasize the clinical need for dual-phase amyloid PET for patients with PD with or at risk of cognitive disorder, which can assess perfusion and amyloid deposition at once. We did not evaluate the performance of kinetic parameters of eFBB PET. In a previous study, a kinetic parameter of amyloid PET (PiB-R1) showed a higher correlation to H_2_O PET compared to early-frame SUVRs of PiB PET (ePIB) on a cortical level in patients with AD. Furthermore, there was a weak positive correlation between mean cortical binding potential and ePIB, but not with PiB-R1, suggesting that the kinetic parameter is robust to amyloid burden^27^. Further studies to figure the contamination of eFBB PET images by amyloid deposition are needed. For spatial normalization of SPECT, there was an error in the co-registration step to MRI images, and the built-in SPECT template in SPM was used instead. The template was derived from HMPAO SPECT, not from ECD. However, the impact of using HMPAO based template would have been small in the spatial normalization.

To conclude, eFBB PET is an imaging test suitable for cortical perfusion evaluation and provides reliable SUVR based on cerebellum references. The FBB PET may serve as one-stop-shop imaging for evaluating amyloid burden and neuronal injury in neurodegenerative diseases, resulting in less medical cost and radiation exposure. The FBB PET may also be helpful to discriminate DLB from PD.

## Methods

### Study design and patients

The institutional review board of our institute approved this retrospective study, and the requirement to obtain informed consent was waived. This study included de novo early Parkinson’s disease (PD) patients who underwent ECD SPECT and FBB PET CT within a 30-day interval from January 1st, 2017, to September 30th, 2020. All patients had T1 weighted brain images for spatial normalization of FBB PET images. Patients with (1) structural abnormality in brain MRI, (2) no available eFBB PET images, (3) no available attenuation-corrected ECD SPECT, and (4) failed imaging spatial normalization were excluded. Demographic information and neurological exam data were collected from medical records. Neurological exams included Korean-Mini Mental Status Examination (K-MMSE), Unified Parkinson’s Disease Rating Scale (UPDRS), and modified Hoehn–Yahr scale. Patients clinically diagnosed as DLB were additionally included to compare the diagnostic performance of eFBB PET and ECD SPECT.

### Image acquisition and reconstruction

A dedicated PET/CT scanner, GE Discovery 710 PET/CT (GE Healthcare, Milwaukee, WI), was used to obtain an FBB PET scan. For eFBB scan, list mode acquisition of 10 min images started immediately after intravenous injection of FBB (300 ± 5 MBq). Late-phase static scans were acquired from 90 to 110 min after FBB injection. Reconstruction of a static image with VPHD-S method (Ordered subsets maximization expectation + Point-spread function reconstruction methods) was applied.

Before and after the injection of ECD, patients rested in a dimmed quiet room. SPECT scan started 30–60 min after intravenous injection of ECD (740 MBq). SPECT acquisition was performed on Siemens Symbia EVO EXCEL SPECT scanner equipped by rotating the camera a total of 180 degrees at the 3-degree interval. Each frame was taken for 20 s. Acquired images were reconstructed on a 128 × 128 matrix using 3D OSEM (8 subsets and 16 iterations) and Chang’s method^28^.

### Image processing

FBB PET and ECD SPECT images were preprocessed using Statistical Parametric Mapping version 8 function (http://www.fil.ion.ac.uk/spm) implemented on MATLAB (version R2020a, http://www.mathworks.com). Early-phase and late-phase FBB PET images of all subjects were coregistered to individual T1-weighted brain MRI images, and those individual MRI images were spatially normalized to a standard Montreal Neurological Institute space. ECD SPECT images were spatially normalized to a built-in perfusion SPECT template from SPM12. The spatially normalized FBB PET and ECD SPECT images were then smoothed with an 8 mm Gaussian filter.

### Image analysis

Two nuclear medicine physicians interpreted late-phase FBB PET images independently (SJK & SH). Final interpretations of images with discordance were made by consensus of two interpreters. Late-phase FBB PET images were visually assessed as previously described^29^. A late-phase FBB PET scan with diffuse FBB uptake in at least one cortical region was considered amyloid positive.

We calculated cortical SUVRs of eFBB PET and ECD SPECT using six candidate reference regions as follows: whole brain (namely, global normalization; GN), central white matter (CWM), pons, whole cerebellum (WC), whole cerebellum with brain stem (WC + B), and cerebellar grey (CG). The VOIs of the latter four references were presented from the Centiloid project. The CWM VOI was derived by extracting voxels with a probability higher than 75% from a probabilistic functional map of the white matter presented from SPM. The Centiloid cortex VOI was used to assess brain cortical signal, which was produced by subtracting the PiB signal of normal control from AD patients, covering the large cortical region of the greatest amyloid load^30^. The regional standardized uptake value ratios (SUVR) were calculated as the mean count of Centiloid cortex VOI over the mean count of each reference VOI for all pairs of early-phase FBB PET and ECD SPECT images. The voxel-wise SUVRs of each image were obtained as each voxel count over the mean count of each reference VOI.

For extraction of the ICNs, a group ICA algorithm to extract coherent network components (GIFT, http://mialab.mrn.org/, GIFT ver 3.0c) was applied. All preprocessed ECD SPECT and eFBB PET images were included in the ICA separately for each modality. The optimal dimensionality number of ICA from each image set was determined based on the assessment of entropy rate. The infomax algorithm was applied to perform ICA. The results of ICA were transformed to Z scores and visualized with the threshold Z > 1.0.

To explore the clinical impact of eFBB PET, we reconstructed VOIs introduced in automated anatomical labeling atlas 2 (AAL2 reference) to 20 cortical segments (Supplementary Table 4). Then we calculated the SUVRs of 20 segments from CG-normalized eFBB PET and ECD SPECT of patients with DLB and PD.

### Statistical analysis

Statistical analyses were performed using GraphPad Prism (version 9.1.2) and SPSS Statistics (v 24). Pearson’s correlation coefficients *r* were calculated for the evaluation of the correlation of cortical SUVRs between eFBB PET and ECD SPECT images. A two-sided *p*-value less than 0.05 was regarded as statistically significant. A voxel-wise correlation test was performed using the Pearson’s correlation test. A paired-samples t-test controlling age was done using SPM8 function for voxel-wise comparison of eFBB PET and ECD SPECT images normalized by CG. We used a threshold of adjusted *p* < 0.05 with options of multiple comparison correction by FDR, and significant clusters included more than 200 voxels for the paired t-test. We performed the Mann–Whitney U test to find VOIs showing significantly different SUVRs between DLB versus PD. Logistic regression models using forward propagation were developed with the SUVRs of the VOIs mentioned above as continuous variables. We performed the ROC analyses to assess the predictive performance of the logistic regression predictive models for discriminating DLB versus PD.

### Ethical approval

All procedures performed in studies involving human participants were in accordance with the ethical standards of the institutional research committee of the Catholic University of Korea, Catholic Medical Center and with the Helsinki declaration as revised in 2013 and its later amendments or comparable ethical standards. For this type of study, formal consent is not required.

### Informed consent

The institutional review board of our institute approved this retrospective study (KC20RISI0857), and the requirement to obtain informed consent was waived.

## Supplementary Information

Supplementary Information.
