# Supplementary material for: Comparison of early F-18 Florbetaben PET/CT to Tc-99m ECD SPECT using voxel, regional, and network analysis
Source: Sci Rep. 2021 Aug 18;11:16738. doi: 10.1038/s41598-021-95808-8 (PMC8373880; doi:10.1038/s41598-021-95808-8)
Supplement: Supplementary file 1 — Supplementary Information. [file 41598_2021_95808_MOESM1_ESM.docx]

**Supplementary data**

**Title of the paper:**

Comparison of early F-18 Florbetaben PET/CT to Tc-99m ECD SPECT using voxel, regional, and network analysis

**The name(s) of the author(s):**

Soo Jin Kwon MD^1^, Seunggyun Ha MD, PhD^*1^, Sang-Won Yoo MD, PhD^2^, Na-Young Shin MD, PhD^3^, Joo Hyun O MD, PhD^1^, Ie Ryung Yoo MD, PhD^1^, Joong-Seok Kim MD, PhD^2^

**The affiliation(s) and address(es) of the author(s):**

^1^Division of Nuclear Medicine, Department of Radiology, Seoul St. Mary′s Hospital, College of Medicine, The Catholic University of Korea, Seoul, Korea

^2^Department of Neurology, College of Medicine, The Catholic University of Korea, Seoul, Republic of Korea

^3^Department of Radiology, College of Medicine, The Catholic University of Korea, Seoul, Republic of Korea

***The corresponding author:**

Seunggyun Ha, MD., PhD.

Division of Nuclear Medicine, Department of Radiology, Seoul St. Mary′s Hospital, College of Medicine, The Catholic University of Korea, 222, Banpo-daero, Seocho-gu, Seoul, 06591 Republic of Korea

Phone: 82-2-2258-1547

e-mail: seunggyun.ha@gmail.com

**Supplementary Figure 1**


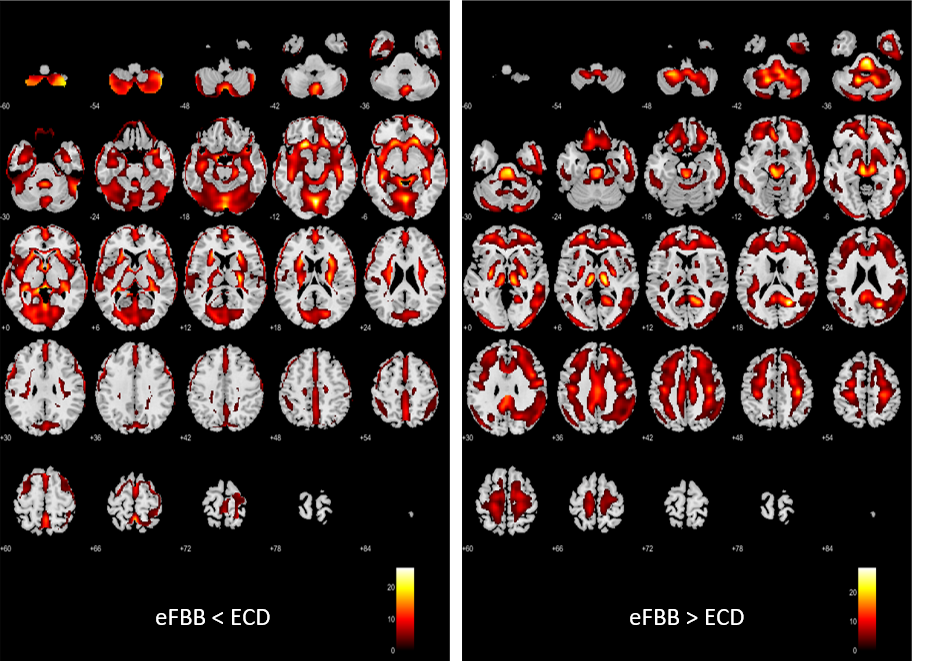


Voxel-wise comparison of eFBB PET and ECD SPECT with normalization by cerebellar grey (p < 0.05, FDR adjusted). The colored areas overlaid on the T1 weighted MRI template show voxels where ECD SPECT showed higher (left) or lower (right) SUVRs compared to that of eFBB PET.

**Supplementary Figure 2**


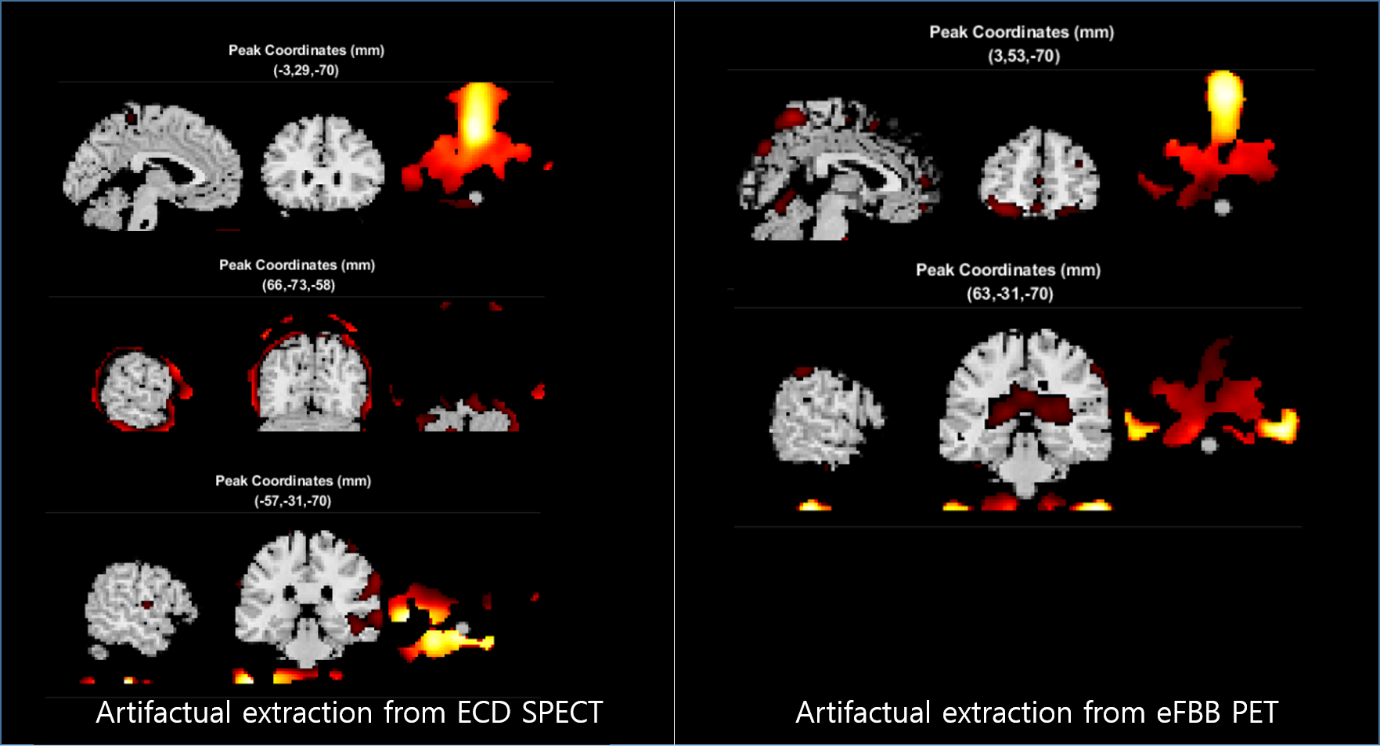


The figure above depicts the artefactually extracted intrinsic connectivity networks (ICNs) from ECD SPECT and eFBB PET. The colored areas show the voxels with Z scores higher than 1.0.

Abbreviations ECD SPECT, Tc-99m ethyl cysteinate dimer single photon emission computed tomography; eFBB PET, early phase F-18 Florbetaben positron emission tomography

**Supplementary Table 1. Characteristics of patients with Diffuse Lewy body disease.**

| **Clinical diagnosis** | **Diffuse Lewy body disease (n=10)** |
| --- | --- |
| Age at diagnosis (median, range) | 79 (71-88) |
| Gender, number (percentage) |  |
| Male | 5 (50%) |
| Female | 5 (50%) |

**Supplementary Table 2. SUVRs of the parietal, occipital cortices, and insula CG-normalized ECD SPECT of patients with DLB and PD.**

|  | DLB | | PD | |  |
| --- | --- | --- | --- | --- | --- |
|  | mean SUVR | SD | mean SUVR | SD | *p* value * |
| Parietal, left | 0.776 | 0.043 | 0.817 | 0.045 | 0.027 |
| Parietal, right | 0.735 | 0.073 | 0.796 | 0.043 | 0.013 |
| Occipital, left | 0.857 | 0.050 | 0.889 | 0.039 | 0.200 |
| Occipital, right | 0.813 | 0.065 | 0.863 | 0.037 | 0.049 |
| Insula, left | 0.777 | 0.084 | 0.848 | 0.056 | 0.019 |
| Insula, right | 0.743 | 0.049 | 0.849 | 0.061 | < 0.001 |

**p* value obtained with Mann-Whitney U test.

Abbreviations CG, Cerebellar gray; DLB, Diffuse Lewy body disease; ECD SPECT, Tc-99m ethyl cysteinate dimer single photon emission computed tomography; eFBB PET, early phase F-18 Florbetaben positron emission tomography; PD Parkinson’s disease; SD, standard deviation; SUVR, Standardized uptake value ratio.

**Supplementary Table 3. SUVRs of the parietal, occipital cortices, and insula of CG-normalized eFBB PET of patients with DLB and PD.**

|  | DLB | | PD | |  |
| --- | --- | --- | --- | --- | --- |
|  | mean SUVR | SD | mean SUVR | SD | *p* value * |
| Parietal, left | 0.764 | 0.063 | 0.795 | 0.048 | 0.128 |
| Parietal, right | 0.707 | 0.088 | 0.777 | 0.042 | 0.014 |
| Occipital, left | 0.851 | 0.054 | 0.868 | 0.057 | 0.297 |
| Occipital, right | 0.817 | 0.073 | 0.854 | 0.053 | 0.166 |
| Insula, left | 0.727 | 0.073 | 0.798 | 0.064 | 0.016 |
| Insula, right | 0.682 | 0.044 | 0.803 | 0.072 | < 0.001 |

**p* value obtained with Mann-Whitney U test.

Abbreviations CG, Cerebellar gray; DLB, Diffuse Lewy body disease; ECD SPECT, Tc-99m ethyl cysteinate dimer single photon emission computed tomography; eFBB PET, early phase F-18 Florbetaben positron emission tomography; PD Parkinson’s disease; SD, standard deviation; SUVR, Standardized uptake value ratio.

**Supplementary Table 4. Reconstructed cortical segments from AAL2 atlas.**

| **New segment name** | **Original AAL2 segment** |
| --- | --- |
| Frontal | Precentral gyrus |
|  | Superior frontal gyrus, dorsolateral |
|  | Middle frontal gyrus |
|  | Inferior frontal gyrus, opercular part |
|  | Inferior frontal gyrus, triangular part |
|  | IFG pars orbitalis, |
|  | Rolandic operculum |
|  | Supplementary motor area |
|  | Olfactory cortex |
|  | Superior frontal gyrus, medial |
|  | Superior frontal gyrus, medial orbital |
|  | Gyrus rectus |
|  | Medial orbital gyrus |
|  | Anterior orbital gyrus |
|  | Posterior orbital gyrus |
|  | Lateral orbital gyrus |
| Insula | Insula |
| Cingulate | Anterior cingulate & paracingulate gyri |
|  | Middle cingulate & paracingulate gyri |
|  | Posterior cingulate gyrus |
| Medial temporal | Hippocampus |
|  | Parahippocampal gyrus |
|  | Amygdala |
| Occipital | Calcarine fissure and surrounding cortex |
|  | Cuneus |
|  | Lingual gyrus |
|  | Superior occipital gyrus |
|  | Middle occipital gyrus |
|  | Inferior occipital gyrus |
| Occipito-temporal | Fusiform gyrus |
| Parietal | Postcentral gyrus |
|  | Superior parietal gyrus |
|  | Inferior parietal gyrus, excluding supramarginal and angular gyri |
|  | SupraMarginal gyrus |
|  | Angular gyrus |
|  | Precuneus |
|  | Paracentral lobule |
| Subcortical-striatum | Caudate nucleus |
|  | Lenticular nucleus, Putamen |
|  | Lenticular nucleus, Pallidum |
| Subcortical-thalamus | Thalamus |
| Lateral temporal | Heschl’s gyrus |
|  | Superior temporal gyrus |
|  | Temporal pole: superior temporal gyrus |
|  | Middle temporal gyrus |
|  | Temporal pole: middle temporal gyrus |
|  | Inferior temporal gyrus |

Abbreviation AAL, Automated anatomical labeling atlas
